# The Design and Implementation of a Custom Platform for the Experimental Tuning of a Quadcopter Controller †

**DOI:** 10.3390/s20071940

**Published:** 2020-03-30

**Authors:** Michał Waliszkiewicz, Konrad Wojtowicz, Zdzisław Rochala, Eulalia Balestrieri

**Affiliations:** 1 Faculty of Mechatronics and Aerospace, Military University of Technology, 00-908 Warsaw, Poland; michal.wa94@gmail.com (M.W.); zdzislaw.rochala@wat.edu.pl (Z.R.); 2 Department of Engineering, University of Sannio, 82100 Benevento, Italy; balestrieri@unisannio.it

**Keywords:** quadcopter, flight controller, regulator tuning, control loop, UAV

## Abstract

This paper describes the development process of the quadcopter-based unmanned flying platform, designed for testing and experimentation purposes. The project features custom-made hardware, which includes the prototype quadcopter frame and the flight controller, and software solutions, such as control loop setup. The article specifies the controller tuning used for the initialization of the flight stabilization system and presents the final results of the quadcopter performance evaluation.

## 1. Introduction

Unmanned aerial vehicles (UAVs) have become a vital part of many industries [1,2]. The usage of the so-called drones has become widespread, which made the various technologies that drive them commercial, prominent, and easily accessible. These factors, in turn, allow for a highly creative approach towards construction of UAVs and their application [3,4].

Hand in hand with the rapid development and increasing number of new UAV implementations and applications, UAV testing has gained increased importance, while at the same time becoming more complex. Different testing solutions have been proposed in the scientific literature, focusing on the tests of the UAV as a whole system or its specific components and systems, including multiple drones. Testing is carried out in all the stages required for UAV development and use. The results provided by the test performed in the intermediate UAV development stages have essential repercussions on the subsequent steps and on the costs to be faced. Particularly crucial is the test carried out in the initial stage of development because, in this stage, the feasibility of the project is decided, and the UAV development costs, times, and difficulties to be faced in the subsequent stages depend on that decision.

UAV testing can involve even new challenges. A good example is the drone testing in the field, wherein the environment is not entirely controllable. The environmental conditions are not repeatable, numerous, and very different from each other. Another problem concerns the UAV regulations that must be met, even though they are continuously evolving and sometimes different depending on the place considered.

Moreover, it should be highlighted that UAV performance strictly depends on the onboard algorithms that can concern its stabilization, control, and navigation. Therefore, it is substantial that these algorithms are adequately tested entirely and faithfully with the target field and application environment. However, this need has not yet found a suitable solution, representing a further challenge for drone testing.

The paper outlines the development of a quadcopter-based unmanned flying platform built using custom UAV hardware and software aimed at providing an efficient solution for custom algorithms in-flight testing in a real environment at the initial and further stages of development.

The paper expands upon several topics already presented in the conference paper [5], including the implementation of the attitude estimation algorithm; hardware design; and execution, the introduction of software functionality, and additional experimental data.

The paper begins with a state-of-the-art analysis followed by a presentation of the platform conception and flight stabilization fundamentals. Section 2 describes the following subsystems of the platform: the airframe’s construction and equipment, and the flight controller’s electronic hardware and software. It goes on with the sample algorithm design, which is developed to be tested with the presented platform. The general information on control loop designing, controller implementation, and attitude estimation is followed by a section highlighting the versatility of the platform as a test tool. Then the details of the sample tuning method are presented, including inner loop and outer loop tuning in the cascade controller. The section ends with a description of the system’s initialization procedure and of the test program. Section 3 presents the result of the tests conducted on the test platform with the sample control algorithms employed. Finally, the results presented in the paper are discussed and the future research direction mentioned.

### 1.1. Systems for Quadcopters Testing

The growing market of small drones involves the development of test solutions for supporting the designing and maintenance of the aircraft.

Among the different existing test-bench solutions, it is possible to distinguish between two main groups. In the first group, benches are providing comprehensive stationary solutions for testing the whole UAV, unmanned aerial system (UAS), which contains UAVs, the ground control station, and accessories, or even the set of UASs. In the second one, there are those designed for testing specific UAV components.

The stationary test-bench is typically designed to test the parameters of the drones mounted in the test-bench [6,7,8]. The drone being tested has to be complete. Therefore, the frame, electronic hardware, and software have to be integrated before the test.

An example of this kind of test bench named DronesBench (University of Sannio, Benevento, Italy) has been proposed in [7,8]. This system allows testing of a UAS as a whole platform but in a controlled environment. In particular, DronesBench measures the attitude of the UAS in terms of pitch, yaw, and roll angles; accelerations; power consumption; and thrust force exerted by UAS [7,8]. A new version of the DronesBench system allowing remote control of the testing procedure over the Internet has also been proposed. With this drone bench, the researchers can control the test simultaneously from various laboratories [6].

Test bench solutions belonging to the second class are mainly focused on components such as the UAV propulsion system, the inertial navigation system, the control system algorithms, and the software, and on a specific related parameter or phenomenon, such as the ground effect or optical payload [5,6,7,8].

An example belonging to the first solution category is the test-bench presented in [9] and designed for the ground effect testing of a quadrotor with eight propellers arranged in four coaxial pairs. A series of experiments have been performed in that research. First, propellers have been tested on a test stand, and then the vehicle has been tested in flight to compute the in-ground and out-of-ground thrusts. In this way, an explicit relationship between the thrust produced by a single propeller while operating in-ground-effect and the thrust out-of-ground effect has been determined as a function of the normalized propeller height above ground [9].

Other critical parameters to be measured in the propulsion system are the thrust forces to the motor speeds, the motor speed response time, and the power efficiency. There is a simple test bench commonly used for the static thrust force measurements. The primary sensor on the bench is an electronic weight balance [10]. The system consists of a rigid rod with a 1° of freedom pivot in the middle. A propulsion system and an electronic balance are fixed on the opposite ends of the rode. Additionally, a tachometer is attached to the drive shaft of the propeller. The motor rotates a propeller and generates the thrust, which is measured by the electronic balance. In this way, the test bench measures the thrust of the propeller depending on the engine speed, which can be applied in the process of the control system design and optimization.

Another example of a test bench is presented in [10] in which the shared driveshaft connects a motor to a load motor. The load motor emulates the operation of the propeller. The more complicated solution also presented in [10] is a balancing test bench with a wind tunnel. This measurement system can determine a relationship between the power consumption of the propulsion system and the thrust in various environmental conditions and different aircraft airspeeds.

A comprehensive UAV testing also requires analyzing it in a real environment. For this reason, the UAV test site-based solutions have been proposed.

An example of a test site simulating the real environmental conditions for a drone along with a test procedure has been presented in [11]. The test site has been designed to test a drone for taking samples of water from a river. The flight can be disrupted by the gusts of wind emulated in the site. The test site consists of a pool which emulates the river and fans for generating wind gusts. There are also test sites simulating the environmental conditions for testing multiple drones and focused on commanding all of them at the same time during a long period [12,13]. The project is based on the simulation software and on the real sandbox for testing ground and unmanned aerial vehicles. That sort of test site has caught the interest of researchers for years. A similar test site had been developed earlier at Stanford University [14]. It is focused mostly on the development and testing of the collision avoidance algorithms for drones. At a similar time, Illinois University researchers presented a successful implementation of the early-stage test site for remote controlling unmanned robots via WIFI [15].

UAV control and navigation algorithm design and testing is a significant challenge due to the more and more stringent performance requirements and the existence of new and very different flight scenarios. For these reasons, researchers’ interest has also been focused on the design and implementation of the UAV testing platform aimed explicitly at UAV control and navigation algorithm testing.

An example of a platform to simulate and test the control system of a tail-sitter UAV has been presented in [16] as an efficient tool for developing the control system. Through the component breakdown approach, a mathematical model of dynamics covering the full angle-of-attack range has been derived along with an environmental model to provide essential information about the environmental influence on the vehicle, such as wind field estimation and wind disturbance control. A commonly used open-source flight controller has been embedded in the proposed framework. The proposed platform has been validated with the typical complete flight scenarios of a tail-sitter, including hovering, forward transition, cruise, and back transition [16]. However, the choice of using the off-the-shelf Pixhawk (controller designed in the team led by Lorenz Meier in Zurich, Switzerland) as a flight controller unit allows only the Pixhawk controller parameters to be tuned. Implementing the user’s algorithm with the original firmware is not possible without replacing all the firmware. Although developing user custom software for Pixhawk is possible, it is a complex and not easy task.

An experimental platform using an evolutionary algorithm for the automatic tuning of the gains of a cascaded proportional–integral–derivative (PID) quadcopter controller has been proposed in [17]. By means of the presented platform, the UAV automatic tuning is allowed, precluding the necessity of a physical operator, domain knowledge, or a model. Tuning to different payloads is also possible [17]. The platform provides an automatic, repeatable, and nondestructive hardware testbed for UAV control, and it has been validated in a sample experiment that automatically tunes a PID controller for quadcopter hover. However, in the research work proposed, the only option is parameter tuning for the embedded PID algorithm. The authors focused less on the issue related to the platform airframe and electronics.

The problem of UAV controller tuning has also been faced in [18]. In particular, the performances of the most used algorithms to tune controllers, and the two new iterative algorithms proposed by the authors, have been evaluated. The performances of the algorithms have been compared using Matlab (MathWorks, Natick, USA) simulations, a quadcopter located in a virtual environment and field tests using an experimental platform consisting of a custom-built quadrotor with an on-board control system, including a MikroKopter (HiSystems GmbH, Moormerland, Germany) flight controller and speed controllers. The proposed iterative algorithms have shown a significant improvement concerning the PID controller tuning performance. However, the tuning based on the results of the in-flight tests has not been considered in the analysis [18].

To sum up, there are many examples of test-benches for drone components and payload testing. Further, there are comprehensive test-sites available for testing complete drones or systems of multiple drones. The researchers have paid particular attention to UAV control and navigation algorithms. As a consequence, some testing platforms have been proposed to solve the problems related to obtaining efficiently and completely satisfactory UAV control. However, UAV dynamics are often complex and sometimes unknown, especially when operating in real environments.

However, there is no platform for testing custom algorithms in-flight, in a real environment and at the initial stage of development. Employing a highly customizable platform and in-depth analysis of the UAV software and algorithms can be carried out, providing researchers with numerous degrees of freedom and high flexibility in proposing and testing new solutions, in a simpler way, in a short time, and without significant financial outlays. The possibility of carrying out in-flight tests in the real environment can provide a better knowledge of UAV dynamics and the factors influencing the performance. Finally, the possibility of carrying out tests already from the initial stage of the UAV development can sharply reduce its costs and required time and significantly optimize the results obtained in the later stages.

### 1.2. Concept

The concept behind the proposed platform is to create a highly customizable, inexpensive, unmanned aerial platform for testing and experimentation. The presented platform delivers all the components to test algorithms and software at the initial stage of development.

The main novelty is an idea of a test platform for hardware-in-the-loop tests of the on-board system algorithms. The platform consists of a frame, propulsion system, electronic hardware, and software that provides diagnostic tools ready for custom algorithm implementation. Stabilization, control, or navigation algorithms can be tested on the platform with the diagnostic tools. With the tools, researchers can monitor the parameters during flight and acquire data for postflight analysis. The tests can be conducted either on the test-stand with limited possible movements of the platform and reduced risk at the initial step of tuning or on the free-flying platform in specified environmental conditions.

The algorithm test starts commonly with the software-in-the-loop simulation. The researcher can move to a hardware-in-the-loop test with simulated sensors when the target on-board computer is ready, and further, to the test on the test-bench when the frame and electronics are prepared. Finally, he can carry out a comprehensive test on a test-site when all the drone systems are operational. Although the systems covering some of the mentioned stages of tests have been developed and described in the literature [6,7,8,9,10,11,12,13,14,15,16,17,18], a platform allowing free-flight tests with the custom user algorithms implemented has not been developed yet. The presented platform offers in-flight tests of the algorithms at the initial stage of development.

Moreover, with this platform, a researcher can also test the software solution in further stages by replacing more and more pieces of software with the new code. In this way, one of the algorithms of stabilization, control, and navigation only can be customized or all of them simultaneously. The frame of the platform is 3D printable and fully reproducible with commonly available 3D printers. In the event of a failure, most parts of the airframe can be 3D printed on-site. Additionally, the frame structure has been designed to be easily transportable. The principle of operation of the folding arms and their construction were designed and tested during the project.

The software of the controller is totally open-source and well documented so that the user can quickly implement his custom control algorithms. The quadcopter is controlled by a custom flight controller operated by the author’s software to achieve optimal flexibility and potential for experimental purposes, backed by the principles presented in [19]. The software provides the embedded in-flight data acquisition tools for in-flight and after-flight diagnostics and analysis. An example process of control algorithms development has been presented to show the consecutive steps of designing, implementing, and testing the algorithms with the platform using embedded diagnostic tools.

### 1.3. Flight Stabilization

The flight controller is an essential part of every quadcopter as its purpose is to maintain the aircraft’s orientation about the ground. This process is often referred to as attitude stabilization and is critical for the aircraft to perform flight successfully [20,21,22]. Every system of this kind must be able to perform the following tasks, necessary to perform flight stabilization:

- Calculate aircraft attitude;
- Receive commands from the pilot (usually via radio receiver);
- Run flight stabilization loop at high frequency;
- Control propulsion.

Reliable acquisition of information about the quadcopter attitude is essential for the flight stabilization to be performed. The attitude can be defined as the relative angular position of two reference frames:

- Quadcopter frame of reference;
- A ground frame of reference.

Both frames have their centers located in the quadcopter center of gravity (i.e., in the sensor location). The ground frame vertical axis is collinear to the Earth gravity vector, while the longitude axis position either is irrelevant or pointing towards the local magnetic north. It depends on the usage of a magnetometer or other means of heading acquisition. The quadcopter frame of reference is tied to its structure, where the longitude axis usually points towards the front of the aircraft.

**Figure 1:**
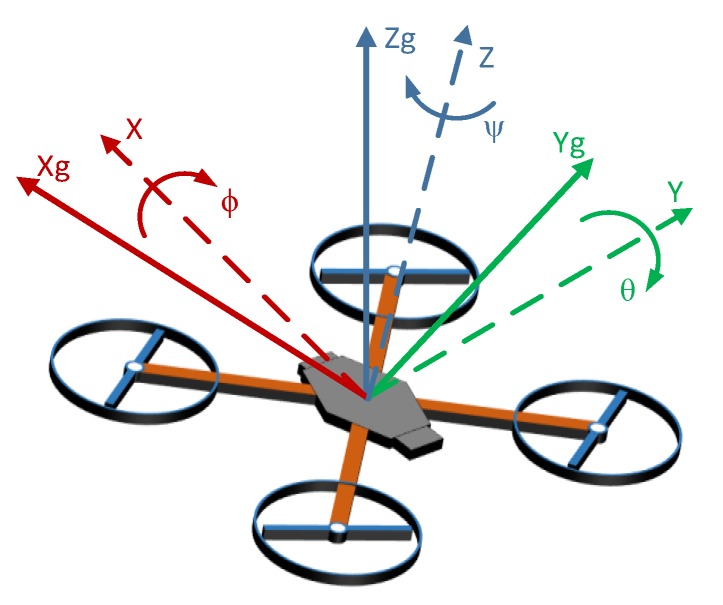
The representation of quadcopter attitude.

The attitude information can be described with a variety of methods such as quaternions or direct cosine matrix [23]. For data presentation, Euler angles—roll pitch and yaw—are most commonly used due to their simplicity. In the context of this project, Euler angles are used to describe the relative angular position of the quadcopter frame of reference (X, Y, Z) concerning the ground frame (Xg, Yg, Zg). The corresponding angular velocities around each axis are represented as $\dot{\phi },\;\dot{\theta },\;\dot{\psi }$, (Figure 1).

The primary purpose of the flight controller is to control the quadcopter motor set to achieve a desirable attitude. This process is formed by comparing the measured attitude information with the set of desired flight parameters, in this case—generated by the pilot ($\phi_{d},\;\theta_{d},\;\psi_{d},\;{\dot{\phi }}_{d},\;{\dot{\theta }}_{d},\;{\dot{\psi }}_{d}$). In the current context, throttle information is not included in the comparison as the implemented solution does not include autonomous altitude control.

In order to acquire the above set of information, the AHRS (Attitude Heading and Reference System) is most often used. It is comprised of a sensor package—IMU (inertial measurement unit) and a processing unit, responsible for data filtering and calculations, with the latter usually being the flight controller’s main microchip. The IMU contains a set of three-axis gyroscopes and accelerometers, sometimes extended with input from a magnetometer. Data from IMU can be used to acquire a complete minimum set of necessary attitude information required for flight stabilization.

Gyroscopes are used to track rapid changes to the quadcopter attitude. Despite them being prone to drift, their resilience to linear accelerations makes them indispensable in-flight applications. In order to counteract the gyro vulnerabilities, accelerometers are used with a twofold role. Firstly, the accelerometers are responsible for maintaining the connection to the ground frame of reference through the measurement of the gravity vector. Secondly, the gathered information is used by the filtering algorithm to diminish the effects of drift from the result of attitude estimation.

**Figure 2:**
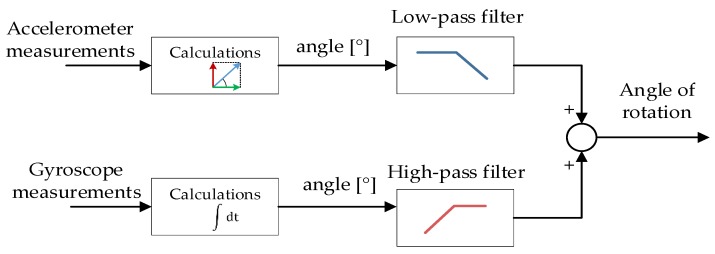
Complementary filter block schematic.

The most optimal way to implement this process takes the form of a “best of both worlds” approach, where data from each sensor is processed in a specific way to provide the most accurate and useful information possible. The process is a fundamental principle of a complementary filter, with its basic block diagram presented in Figure 2.

Attitude derived from the accelerometer is processed using a low-pass filter to minimize the presence of vibration-induced noise in the result. In contrast, the angle derived by integrating the gyro is processed using a high-pass filter, due to the presence of drift. Such a solution can be written down and implemented with the following formula:

(1) $${\mathrm{angle}}_{est}=\left(1-\alpha \right)*\left({\mathrm{angle}}_{est\_prev}+\int_{t-1}^{t}gyro_{dps}*\mathrm{dt}\right)+{\;\alpha *\mathrm{angle}}_{acc},\;\;0<\alpha <1$$

where: ${\mathrm{angle}}_{est}$—is the estimated orientation angle for a given rotation axis, ${\mathrm{angle}}_{est\_prev}$—estimated orientation angle from the previous iteration, gyro—are the gyroscope readings for given axis (scaled degrees per second), ${\mathrm{angle}}_{acc}$—the orientation angle calculated based acceleration vector measurement, α—is the weight coefficient, used to tune the filter.

Like almost any kind of data processing, such a filter must be run continuously in a loop to work correctly. During each iteration, the weight coefficient, here represented by α, manages the amount of contribution of each sensor in the result. Finding the optimal value of α is key to achieving the most promising results out of this fusion technique. As a rule of thumb, the optimal α value is that which would allow making the lowest possible accelerometer contribution, while being sufficient to compensate for gyro drift. If this contribution were too low, the angle calculated by the gyro would converge too slowly onto the accelerometer-derived angle, and drift would likely cause the estimation error to increase over time. On the other hand, with α value being too high, due to excessive accelerometer contribution, the effects such as vibrations or undesirable linear accelerations would cause issues too significant to consider the filter a reliable source of information.

This technique, while useful in its simplicity, has its limitations uniquely when combining corresponding data from more than two sources. Still, it can be viewed as a primary archetype for much more advanced solutions such as Mahony’s and Madgwick’s filters [24], the latter of which was ultimately integrated into this project flight controller software as means of attitude determination.

## 2. Materials and Methods

### 2.1. Platform Construction

**Figure 3:**
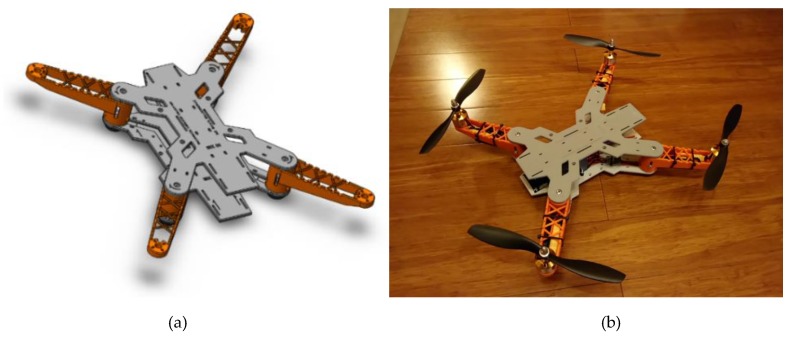
(**a**)—The 3D model of a quadcopter frame design [5], (**b**)—fully assembled quadcopter.

The quadcopter frame is made out of 3D-printed components made using FDM (Fused Deposition Modeling) method. Each element was designed beforehand using a Computer-Aided Design (CAD) software (Figure 3a) [20]. This approach allowed us to achieve the type of frame that fully meets the project requirements, most prominent of which are related to high capacity and customizability. The frame geometry was made to be fully compatible with the preselected off-the-shelf electronic equipment with a limited degree of miniaturization, mostly intended for hobby usage, hence the emphasis on high capacity.

The frame structure was designed with attention to mass distribution, with the final components layout already present during the early stages of development. Another valuable aspect behind the usage of 3D printing was access to spare parts. In case the frame would suffer any significant damage during its use, newly printed parts could replace the broken components in a relatively short amount of time and at a low cost.

The completed quadcopter (Figure 3b), utilizes 10-inch propellers mounted on four ABC-Power A2212 1400 KV brushless motors, each of which is rated for 5.884 N of thrust. The frame opposite corner motor distance measures at approximately 400 mm.

**Figure 4:**
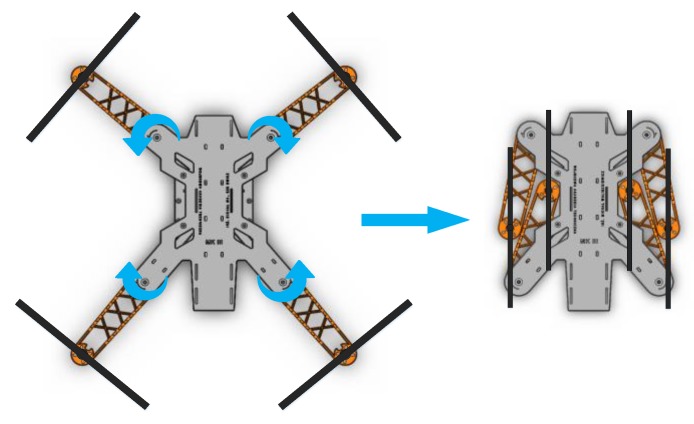
Operating principle of the frame foldable arms [5].

The frame portability was increased substantially by the inclusion of a foldable arms mechanism that uses a simple spring-based locking solution that allows the arms to be folded manually by rotating them inwards (Figure 4). This feature enables the quadcopter to be conveniently packed for transport or storage without the need for removing the propellers.

The prototype of the frame was printed using PLA (polylactide). Still, the material was later replaced with PETG (polyethylene terephthalate glycol-modified) using a 40% rectangular infill to improve further the structural integrity and the quality of the printed parts, due to the latter showing better adhesive properties.

The minimal set of electronics includes the flight controller, radio receiver, and power distribution board, with some space left available for future upgrades such as additional sensors or a camera. The takeoff mass was determined to be about 1.3 kg, which is comparable to medium-sized UAVs available on the market.

### 2.2. Flight Controller—Hardware

The developed prototype flight controller was intended to be fully open for both hardware and software modifications. With that in mind, components selected for its construction were chosen with an emphasis on accessibility and configurability. It was deduced that for the system to be equipped adequately for experimental purposes, some means of on-board flight data transmission and recording would have to be present.

After examining the solutions available on the market, the ESP32 series microcontroller [25] was picked as the central processing unit for the flight controller. The chip features an integrated wireless communication module, which allowed the implementation of a fully functional, close-range telemetric data transmission system, without the need for 3rd party equipment. Furthermore, the chip uses a 32-bit dual-core processor, 4 MB of FLASH, and 520 KB SRAM memory blocks and numerous data-bus interfaces: 4 SPI, 2 I2S, 2 I2C, 3 UART, and a CAN bus controller [26]. In terms of software execution, the module offers multi-threading capabilities through the use of the FreeRTOS operating system, and the support of core Arduino libraries.

The initial flight controller prototype was provided with a basic MEMS sensor package. The choice came in the form of MPU6050 by InvenSense, which already proved itself in numerous UAV applications. The sensor combines an integrated 3-axis accelerometer and gyroscope modules that provide the data necessary for accurate attitude estimation.

**Figure 5:**
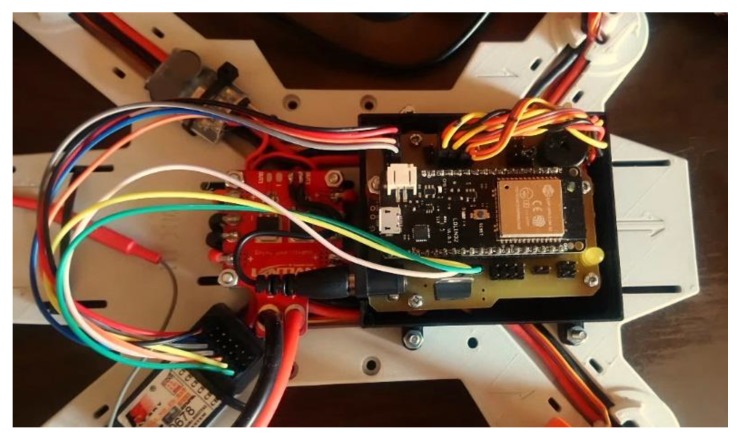
The prototype flight controller mounted on board [5].

In order to uphold the notion of hardware accessibility, the above components were integrated using off-the-shelf modules designed for prototyping. The ESP32 comes with Lolin32 prototyping made by Wemos, while the MPU6050 was supplied on a small circuit board manufactured by DFRobot. The flight controller was put together using a custom PCB (Figure 5).

The board provides all the necessary connections and mounting points for electronic components and a buzzer, led indicator, and a connector for additional power supply. A simple 3D-printable case was designed as well to provide the board with proper protection from external damage.

### 2.3. Flight Controller—Software

The software was written in C++, with most of the hardware functionality being accessed through the use of Arduino core libraries, provided by Espressif. The code was developed using PlatformIO IDE plugin for Visual Studio Code.

The program’s main loop is executed at a frequency of 200 Hz. It consists of typical flight controller functionality, as described in [27], which includes sensor reading, attitude calculations, control loop pipeline, and motors control. For debugging and ad-hoc configuration, the software contains various subroutines accessible through both USB and wireless interfaces, which allow for the modification of the device operational parameters without the need for re-uploading new firmware. However, if necessary, the module is capable of wireless firmware uploads, which is not common in embedded devices.

The wireless telemetry capabilities are provided via an integrated Wi-Fi module that can function both as an access point and a client for the host station and is compatible with virtually any modern computer and smartphone device. The telemetric data can be streamed on-demand, with the use of the UDP (User Datagram Protocol) protocol—the solution chosen mostly due to its high performance and ease of use. UDP does not provide handshaking before or after the data exchange, which has a positive impact on the overall bandwidth. On the other hand, TCP (Transmission Control Protocol) is a slower but more reliable counterpart because it verifies the retrieval of each packet by the recipient device.

### 2.4. Control Loop Design

The flight controller was initially developed with a basic sensor package in the form of a single inertial sensors module. Therefore, in the initial version of the control algorithm, the control loop is limited to attitude stabilization based solely on the pilot’s input in reference to the estimated attitude obtained from AHRS.

A handful of designs was considered for the example feedback loop setup. The simplest was direct angular stabilization in reference to the ground. The most advanced was linear–quadratic regulator (LQR). The final choice came in the form of cascaded PID (Proportional Integral Derivative) feedback loop, which was considered the viable option to implement, given the project already present complexity. Such solutions are proven to be very effective in multirotor applications [27,28,29,30,31].

**Figure 6:**
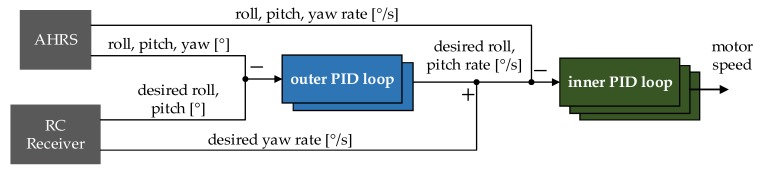
Simplified control loop diagram [5].

The control loop design, as mentioned above, provides a dual set of PID regulators for roll and pitch axis, respectively, consisting of the “inner” and the “outer” loop control loops. The vertical yaw axis is stabilized individually by one controller belonging to the inner loop (Figure 6). In this case, the rotation speed around the yaw axis is directly proportional to the position of the control stick on the radio controller.

The outer PID loop function is to calculate the optimal target rotation speed in the given axis that needs to be applied to decrease the input error. Reducing error is performed by subtracting the desired angular position in the given axis, provided by the pilot, from the values estimated by the AHRS. The calculated angular velocity is then relayed to the inner loop regulator as one of its inputs.

The inner loop is responsible for controlling the motors, based on the information received from the previous PID set. Similarly, the calculated angular velocities are compared with the readings obtained from the gyroscope module. The computed output signal is then used to generate the pulse-width modulation (PWM) wave with a given duty cycle, which drives the brushless DC motors at the desired speed.

In other words, the inner loop stabilizes the quadcopter angular velocity with the use of gyro readings since the outer loop sets a target to the ground in an angular position desired by the pilot. Such setup is potentially effective for fast rejection of disturbances and reduction of their impact on the rest of the control loop [32].

The inner loop output is comprised of three signals, each of which corresponds to the given rotation axis. These signals need to be appropriately combined (mixed) to generate the appropriate output for each motor.

**Figure 7:**
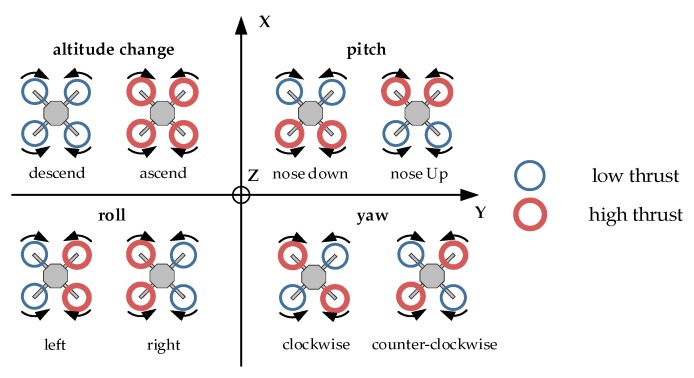
Basic principles of quadcopter maneuvering.

**Figure 8:**
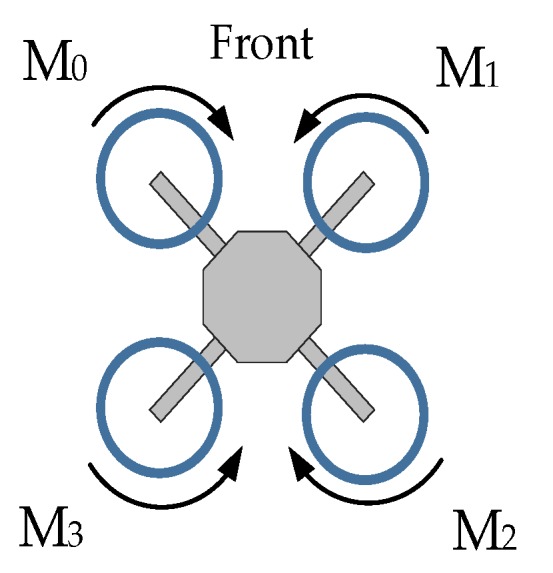
Motor markings.

The quadcopter momentary attitude variations can be viewed as the sum of rotations around its roll, pitch, and yaw axis. Since the system must be able to monitor and act upon all three rotation speeds simultaneously, the output signal for each motor needs to be a combination of all three output values generated by the inner loop regulators: $R_{rate\;out}$ (roll axis), $P_{rate\;out}$ (pitch axis) and $Y_{rate\;out}$ (yaw axis). The math behind the process is based on the quadcopter flight behavior characteristics (Figure 7) and the position of a given motor (Figure 8).

For example, for the aircraft to rotate around the longitudinal axis (X—roll), the motors on the one side of this axis must increase the generated thrust, while at the same time, motors on the other side reduce power. Therefore, the roll output for the motors on the one side must be negated. Similarly, to start rotating around its vertical (Z—yaw) axis, the motors spinning in one direction must generate more thrust while the motors rotating in the opposite direction should lower their speed to generate the asymmetry in the overall momentum applied to the frame. For the project, the motors were numbered in the order starting clockwise from the front left motor—$M_{0}$, to the rear left motor—$M_{3}$.

By using the above markings, the proposed signal mixing formula can be presented by the following set of equations:

(2) $$\begin{array}{c} M_{0}=T_{g}+R_{rate\;out}+P_{rate\;out}+Y_{rate\;out}\\ M_{1}=T_{g}-R_{rate\;out}+P_{rate\;out}-Y_{rate\;out}\\ M_{2}=T_{g}-R_{rate\;out}-P_{rate\;out}+Y_{rate\;out}\\ M_{3}=T_{g}+R_{rate\;out}-P_{rate\;out}-Y_{rate\;out} \end{array}$$

where: $M_{0}\ldots M_{3}$—motor signals, $T_{g}$—pilot’s throttle signal, $R_{rate\;out}$, $P_{rate\;out}$, $Y_{rate\;out}$—inner loop output for roll, pitch, and yaw controllers.

The mixing equation presented above, albeit very basic, is what essentially makes it possible for the quadcopter to autonomously decrease its attitude error with the use of its four motors, which in turn makes the aircraft controllable.

Overall, the control loop operation can be configured by a total of 15 parameters listed in Table 1.

The control loop contains five individual regulators—three belonging to the inner loop and two residing within the outer control loop, with the yaw axis being stabilized using a singular rate controller (Figure 6). With each one having a specific P, I and D gain values assigned to it. Finding the optimal values for these gains was a crucial task that required establishing a tuning method that would provide freedom of experimentation while maintaining proper safety measures.

The proposed method takes from the heuristic Ziegler–Nichols approach, by which the regulator gains are acquired with the use of measuring the oscillatory system response. The measured period of the system response is compared to the standardized tabular values upon which the gains are calculated.

### 2.5. Controller Implementation

It was decided that proportional gain would be separated from integral and derivative blocks to achieve distinctiveness between the contributions of each gain. The following equation describes the controller in its discrete form:

(3) $$u\left(n\right)=K_{p}e\left(n\right)+\frac{\Delta T}{K_{i}}\overset{n}{\underset{j=0}{\sum }}e\left(j\right)+\frac{K_{d}}{\Delta T}\left[e\left(n\right)-e\left(n-1\right)\right]$$

where: $K_{p}$—proportional gain, $K_{i}$—integral gain, $K_{d}$—derivative gain, n –sample number, u(n)—output signal, e(n)—error, ΔT—sampling period.

**Figure 9:**
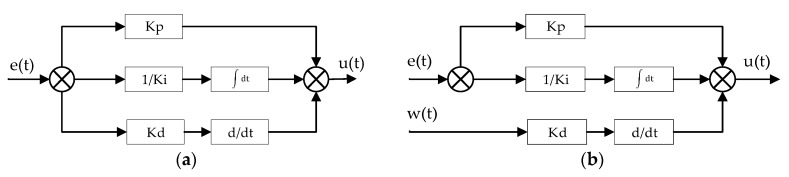
Comparison of basic PID regulator types. (**a**)—the classic approach with single error input, (**b**)—regulator with derivative gain input connected directly to sensor measurements (used in the project), where e(t)—input error, w(t)—sensor measurement, u(t)—regulator output.

Furthermore, the regulator operates in the digital domain. Therefore, the controller was designed with its derivative gain input being connected directly to the sensor measurement data—w(t) instead of the classical approach where the regulator is provided with a single error input—e(t), as presented in Figure 9. It is a form of protection against rapid increases of derivative value due to signal spikes in the controller input.

Interestingly enough, such undesirable conditions were accidentally encountered during software development, long before the flight trials could begin. The cause was discovered within the on-board radio receiver, used as means of remotely steering the aircraft. The device used in the project was programmed to update its output registers with a frequency of 50 Hz, which is a quarter of the frequency of control loop execution (200 Hz). It caused the derivative gain to generate a spike in its output every time the receiver would modify its output signals, which was the reason why the above solution was implemented [33,34].

The set of controllers was implemented in the form of a custom-written class library containing numerous functions that allow for adjusting the control parameters of each regulator individually at any time during the control loop operation. The example code can be found in Appendix A. Both the inner and the outer control loop regulators have the same structure, with the difference being the input and gain settings provided separately, depending on the controller function.

**Figure 10:**
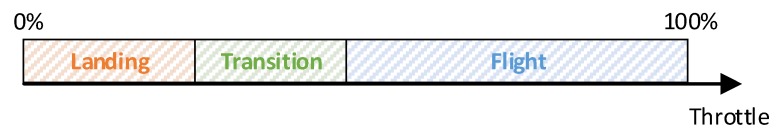
Anti-windup throttle range.

Anti-windup functionality was implemented as well, with its logic being run outside the main class. This functionality is controlled by a process that tracks the throttle value set by the pilot. Through a set of logic, tests determine the current anti-windup setting by comparing the throttle value to a specified set of boundaries. The throttle range was divided into three sections. The lower part intended for landing, the transition zone, and the flight range, which ends at a 100% setting (Figure 10). The transition zone works as a buffer. By operating similarly to a hysteresis loop, it prevents the safeguard from activating unexpectedly during flight. The boundary values for the transition range were estimated based on the thrust range depicted in the datasheet of the motors and then adjusted after the first test flight.

The anti-windup is switched on as default and remains active until the throttle value goes outside the lower range. Shifting the throttle value in or out of the transition zone triggers the anti-windup module to change a mode of operation. Transition into the flight range indicates takeoff in which case safeguard is turned off, making the controllers fully operational. The lower range is used mostly for landing. In that instance, the anti-windup is turned on, the cumulated errors are nullified, and integral gains are left disabled for all controllers.

### 2.6. Attitude Estimation

**Figure 11:**
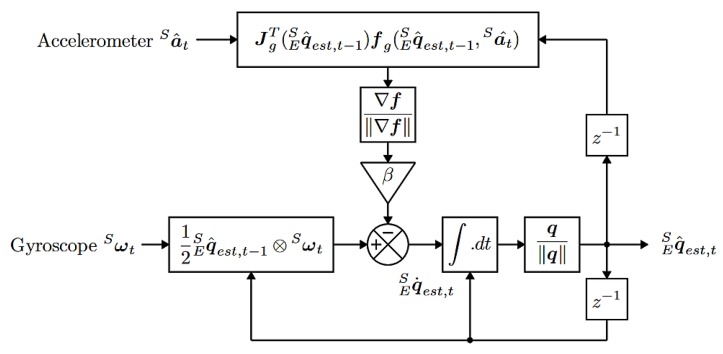
Block diagram of Madgwick’s filter in basic IMU (inertial measurement unit) application [35].

As for the method by which the quadcopter attitude is calculated, while the MPU6050 does provide an onboard proprietary attitude calculation algorithm due to its limited customizability and various software-related issues, an alternative solution was used instead. The implemented algorithm is based on S. Madgwick’s filter, more comprehensively described in [35]. The algorithm outputs information about the quadcopter attitude about the ground in the form of Euler angles, using the measurement data provided by the inertial sensor (Figure 11).

This particular filter was chosen due to its availability in the form of a C-compatible library and a widely proven performance record. Moreover, the filter is well suited for aircraft applications as it allows for the addition of magnetometer in the future development to aid in the quadcopter heading acquisition.

The filter was supplied as a library with two operating modes available—with or without a magnetometer contribution. The algorithm can be configured during initialization by providing it with two operational parameters: sampling frequency and beta coefficient—β, with the latter one requiring further explanation.

Madgwick’s filter utilizes quaternions to describe the sensor (S) orientation relative to earth (E)—qESest, t. This method uses the so-called gradient descent algorithm to remove the impact of the gyroscope error on the computation resulting. Gyroscope measurements form the basis of attitude tracking, while subsidiary sensors such as an accelerometer and also magnetometer are used to estimate the gyroscope error. The basis for this process can be written as the following formulas:

(4) qESest, t=q^ESest, t−1+q˙ESest, tΔt

(5) q˙ESest, t=q˙ESω, t−βq^˙ESϵ, t

Quaternion q^˙ESϵ, t represents the estimated gyroscope error calculated by the gradient descent algorithm. The filter uses data gathered from the accelerometer with the optional addition of a magnetometer to calculate the estimated gyroscope error in the form of a quaternion derivative. This derivative is then used to compensate for the gyroscope error by removing it from the gyroscope measurement quaternion q˙ESω, t as it is in equation 2.5. The presence of β enables us to manually fine-tune this process to achieve the best possible performance out of the available hardware. The author proposes that the β coefficient may be calculated based on mean zero gyroscope measurement error—${\tilde{\omega }}_{\beta }$ using the following formula:

(6) $$\beta =\sqrt{\frac{3}{4}}{\tilde{\omega }}_{\beta }$$

Although the method of gyroscope drift nullification in Madgwick’s filter differs significantly in its inner workings compared to a complementary filter by experimenting with different β values, one might notice that β has similar properties to that of an α coefficient of a complementary filter. With β set too low, the drift correction becomes suppressed to the point of inefficiency, and the filter becomes unusable due to continuously increasing error caused by gyro drift. When the β value is set too high, the filter overcompensates for the drift and output unreliable results. Based on numerous tests with different sensors, it was deduced that typical β values range between 0.02 and 0.10. Cases that required higher β values were resolved by improving the sensor offsets calibration technique. During the following tests, the filter was operating with β = 0.05.

### 2.7. Platform Versatility

The proposed platform has been designed to be versatile in terms of software and hardware configurability alike. The usage of open-source solutions and custom quadcopter frame design makes the platform suitable for custom modifications, allowing the inclusion of new components and control solutions to the aircraft’s current feature set.

The frame itself, being designed in CAD software, can be easily modified to feature any form of sensors, by suitably changing its source files. Moreover, it is possible to alter a single subpart of the frame, for instance, the lower deck, to feature custom mounting points for a new type of sensor/device while maintaining compatibility with the default components. It is also possible to modify the arms to feature the mounting points for new, more efficient motors. With the current accessibility of 3D printing technology, the freedom of frame geometry customization is unmatched in comparison to any mass production solution.

The flight controller has been designed to have a multi-use potential being achieved through the software design and putting the emphasis on the high openness and customizability requirements. The code is fully modifiable as the software functionality can be changed in all aspects, beginning from the primary control loop settings to rewriting the entire subroutines.

Code portability is assured through the use of Arduino libraries as its hardware abstraction layer. This portability makes the control pipeline executable on other common microcontroller-based hardware platforms. The software was developed with a “block design” approach. In essence, subroutines are grouped based on their functionality, which makes it convenient to add new features without significantly impacting the overall code structure. In its current form, the software features a simple, yet efficient control loop design, which makes it perfect as a learning platform, while being open for improving upon the existing hardware and software solutions.

Lastly, due to the way the platform has been conceived initially, the solution fosters creativity above all. The platform seems to be suitable for experimenting with a nonconventional approach to traditional ideas when the use of commercial equipment is either not viable or too restrictive.

### 2.8. Tuning Method

Since the Ziegler–Nichols (Z-N) method can only be applied to a moving system, a decision was made to construct some form of a test-stand for limiting the degrees of freedom of the quadcopter.

The test-stand was made as a rigid metal frame mounted on top of a square wooden platform. The frame is equipped with two hooks used to mount the quadcopter between the two poles using wire. The fixing enables the quadcopter to rotate around its longitudinal axis without obstruction while keeping its center of mass in a fixed position. The adjustment of the control loop parameters during the procedure was made via the already mentioned subroutines contained within the flight controller software, which were accessed wirelessly using a dedicated terminal application.

The tuning process was introduced with the focus on the following set of assumptions:

- The acquired gains are considered as initial values with the expectation that further adjustment has to be conducted in flight,
- The Ziegler–Nichols tuning method is used to tune the inner control only,
- The outer loop is tuned manually with the proportional gains being adjusted first,
- The gains acquired for the roll axis is initially applied to the quadcopter pitch axis, further adjustment to follow if necessary.

The control loop gains affect the drone’s airworthiness and its behavior concerning the pilot’s commands. Therefore, the final parameters’ values can be chosen after the first set of flights is completed, and it depends on the pilot’s preferences.

To achieve the best results, we decided that the influence of each gain of the inner loop controllers would be adequately studied to gather necessary information about its impact on the controller’s overall performance. The tests were performed using the already mentioned test-bed, with the cascade of roll axis controllers being the object of the experiments. The outer loop proportional gain setting was left intact ad matched those resulting from the Z–N method. The pitch and yaw controllers were left disabled for the duration of the test and throttle was limited to 40% for safety. The proportional and derivative gains were tested by applying a 35°-step signal in place of the pilot’s roll axis input to the controller using a software subroutine activated with one of radio receiver auxiliary channels.

In contrast, the integral gain was tested during hover flight. After each test, a given gain factor was incrementally increased, and the procedure was repeated. Data were recorded using the ESP32 as a wireless telemetry module, and the PC application was used for logging the data. The results are presented in Section 3.1.

### 2.9. Inner Loop Tuning

**Figure 12:**
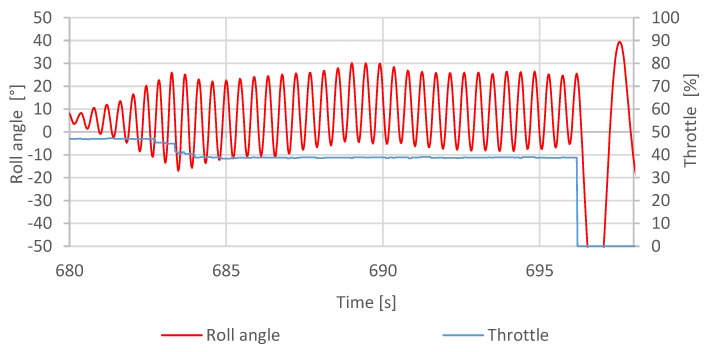
Data gathered during the Z–N (Ziegler–Nichols) tuning procedure—$K_{u}=1.1$ [5].

The inner loop tuning procedure was performed using the already mentioned test-bed. The regulators responsible for lateral and vertical stabilization were left disabled for the duration of the process. The measurement recording began after applying 40% of the entire throttle range to the motors and giving the quadcopter a gentle push to shake it out of balance using the stick on the radio receiver (Figure 12).

The data were acquired using the aforementioned integrated telemetry system and a simple PC application running on the receiving end.

The proportional was slowly increased using the text commands sent to the flight controller via the text terminal application until the ultimate was defined as l $K_{u\;roll}=1.1$ with the registered oscillatory period of $T_{u\;roll}\approx 400\;ms$. These values were applied to the tabular data [36] to calculate the controller gains using the following equations:

(7) $$\begin{array}{c} K_{p\;rate\;roll}=K_{p\;rate\;pitch}=0.6*K_{u\;roll}=0.66\\ K_{i\;rate\;roll}=K_{i\;rate\;pitch}=0.5*T_{u\;roll}*K_{p\;rate}=0.14\\ K_{d\;rate\;roll}=K_{d\;rate\;pitch}=\\ =0.125*T_{u\;roll}*K_{p\;rate}=0.034 \end{array}$$

### 2.10. Outer Loop Tuning

While the inner loop tuning needed to be performed during its operation, due to its direct connection to the propulsion system, the external loop gain could be computed numerically. The calculation is an estimation of the maximum desired rotation speed resulting from the pilot’s input and applying it to a simple multiplication. The regulator proportional gain output is based on this estimation.

The initial values for the outer loop proportional gains were set at $P_{angle\;roll}=P_{angle\;pitch}=4.0$.

Based on the already described outer loop functionality, this produces a relation where 45° difference between measured and desired angles results in the target angular speed of 180 °/s. This setting was further verified in flight and determined to be sufficient. Furthermore, the remaining I and D gains were deemed unnecessary, with the steady error nullification and derivative response already present in the inner loop regulators.

At that point, the quadcopter possessed the ability to perform basic hover flight, with the vertical axis stabilization remaining unadjusted. The inner loop yaw axis proportional gain was tuned experimentally, between flights. The most optimal gain was quickly determined to be $K_{p\;rate\;yaw}=3.0$. Similar to the outer loop, the remaining gains were left disabled. The gains can be left disabled since the quadcopter propellers demonstrate natural dampening effects, which diminish any disturbances affecting the quadcopter rotation around its vertical axis.

### 2.11. Sensor Initialization

Sensors were initialized with the use of a particular subroutine embedded within the software. Each axis was initialized with offsets calculated by averaging a sum of 100 measurements taken over 10 s, during which the quadcopter remained immobile in a horizontal position. The offset values were then saved to the microcontroller non-volatile memory so that they could be loaded during boot-up. The measurements were taken in stable conditions in a static room temperature of 29 °C. The calculated offsets are presented in Table 2.

After the initialization, a series of tests were conducted to verify the validity of calculated offsets and examine the behavior of sensor readings over more extended periods. The first test has been taken for 25 min to observe any deviations from the initial values. It consisted of taking attitude measurements and IMU sensor readings, including the temperature of the sensor. The environment conditions matched the conditions during the initialization. The results are presented below in Section 3.1.

**Figure 13:**
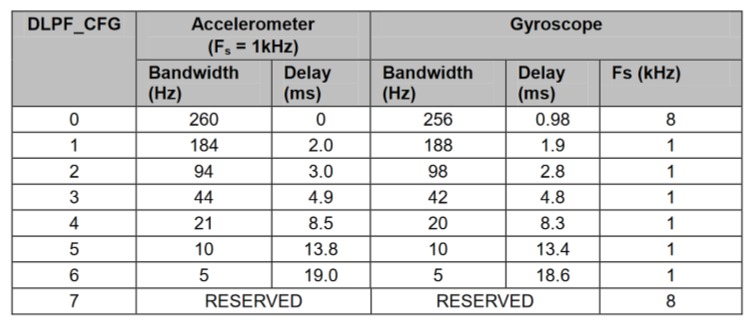
Inertial sensor’s digital low pass filter register map [37].

The sensor package was reconfigured to increase its resilience to vibrations introduced by the motor operation as well. The integrated digital low-pass filter was put in use to counter the influence of high-frequency disturbances on obtained sensor measurements. This feature turned out to be very convenient as it makes software filtering redundant, thus reducing the necessary microcontroller workload. Cutoff frequencies of 44 Hz and 42 Hz were chosen for the accelerometer and gyroscope, respectively, based on the sensor documentation (Figure 13).

**Figure 14:**
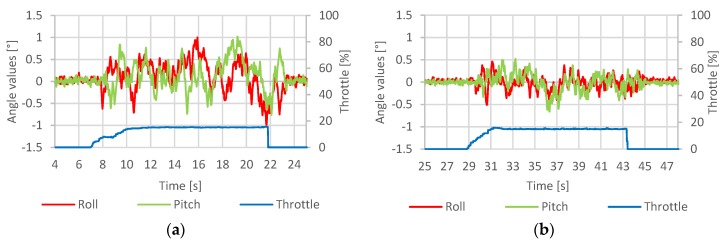
Effects of motor vibrations on the estimated attitude: (**a**) no filtering, (**b**) low-pass filtering with 44/42 Hz cutoff frequencies [5].

The setting was later tested during quadcopter operation. The test was done by mounting the quadcopter in a fixed position and taking measurements of the flight controller estimated attitude angles while applying 15% of thrust to the engines for a specific time. Despite the flight controller housing containing a set of spring-based dampers upon which the PCB was mounted to the frame, the effect of vibrations on attitude estimation exceeded the initial expectations (Figure 14a).

Further tests showed that the effects of vibrations decreased proportionally as the cutoff frequency was lowered. Ultimately the 44/42 Hz frequency set was chosen as the optimal setting, as it demonstrated a compromise between the filter efficiency and introduced delays in sensor response (Figure 14b). The improvement in accuracy is visible, with the deviation range being reduced to ±0.5° about the real value of 0°. The presented register settings provided the best possible results during the tests and were implemented into the AHRS to improve its accuracy.

### 2.12. System Evaluation

A series of tests have been performed to check the quality of the designed system. All the presented tests were taken on the test platform using embedded software tools for tracing and acquisition of the parameters during operation. First, the sensor offsets were examined for their effectiveness during the system’s operation. The purpose of the test was to determine a drift of the sensors and measurement systems in time. In this test, the platform was fixed in a stand, and the on-board system has been started for 20 min. Since the platform was fixed, no input was exerted. The output parameters measured were measurement system temperature, angular speed indicated by gyroscopes, acceleration indicated by accelerometers, pitch, roll, and yaw angles calculated by the inertial measurement unit. Inertial sensors, i.e., a gyroscope and an accelerometer reading, were examined to verify the calculated offsets over the prolonged activity (Section 3.1).

The controller tuning methods have been tested consecutively for tuning in the inner and outer control loops. Separately, the system has been examined against various gains values in each axis since these parameters are considered crucial for successful control (Section 3.2). The purpose of this test was to determine the step response of the controller to the input angular movement exerted consecutively in each axis with various gain values. In this test, the platform was fixed to allow angular movement around one axis in one test only. The input was an angular movement of 35 degrees exerted as a step consecutively around axis x and y. The input was set to 35 degrees because this is an angular boundary position of the drone during its operation. The output was a response from the controller.

Next, the dynamic test of the controller response in each axis with various values of the $K_{i}$ the coefficient was performed. The purpose of this test was to determine a response of the controller to the dynamically changing demanded angular position command with various values of the $K_{i}$ coefficient. The input was a demanded value of the angle in a specified axis. The input was changed randomly by the operator via a radio controller. The output was an angle position in this axis of the drone.

On the pre-tuned controller, a hover stability test and a responsiveness test have been carried out to check the performance of the system. The purpose of this test was to determine the response of the controller to the natural disturbance during hovering. The shape of the response chart is connected to platform stability. Hover stability was examined in an open area with no wind. After takeoff, the quadcopter was placed at an altitude of around 3 m, after which the telemetry recording was started. Due to the flight controller lacking the ability to maintain its position autonomously, minor periodic course corrections were manually applied. Despite this drawback, it was possible to record a 20-s long autonomous flight during which the flight controller attempted to stabilize the quadcopter horizontally. During that time, the quadcopter remained within a radius of two meters from its takeoff location. The input was a demanded value of an angle in a specified axis. The demanded value was set zero degrees as for the hover of the drone. The output was an angle position in this axis of the drone.

The system response time was verified in similar environmental conditions by applying a series of rapid maneuvers to register any significant delays between the pilot’s commands and their execution by the flight control system. The input was a demanded value of an angle in a specified axis changing rapidly. The input was changed randomly by the operator via a radio controller. The output was a delay of the angle position change in this axis of the drone (Section 3.3).

The wireless telemetry system was mostly tested for its maximum operational range capabilities. The purpose of this test was to determine the maximum efficient range of the test platform telemetry. The experiment was conducted in an open area, with the quadcopter and laptop maintaining a direct line of sight between each other for its entire duration, with an active connection. The terminal application was used to register and log the number of received telemetry frames in a given second. The procedure involved carrying the quadcopter and going away from the start point at a steady speed until the distance of around 300 m was reached. The time from the beginning of the test was also measured. The input was a distance between the flying platform and the ground control station. The initial value of the input was set to zero meters, and it was being increased to 180 m because, at this distance, a number of successfully transferred data packets diminished significantly. The output was a number of data packets transferred in time. We performed the test to process the gathered data afterward in the way to synchronize the incremental changes in the distance with the recorded log using a unified time scale (Section 3.4).

## 3. Results

### 3.1. Sensor Offsets Evaluation

**Figure 15:**
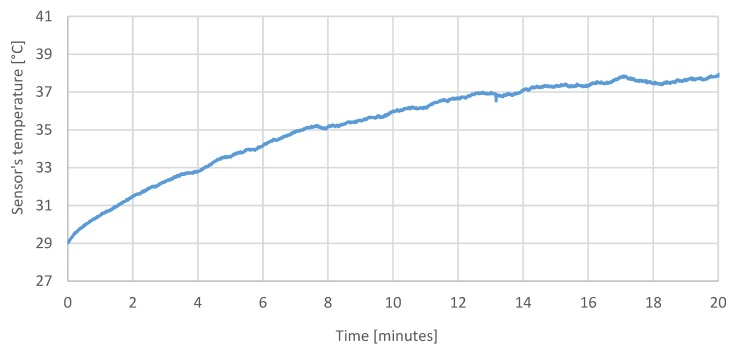
Sensor temperature during the test.

**Figure 16:**
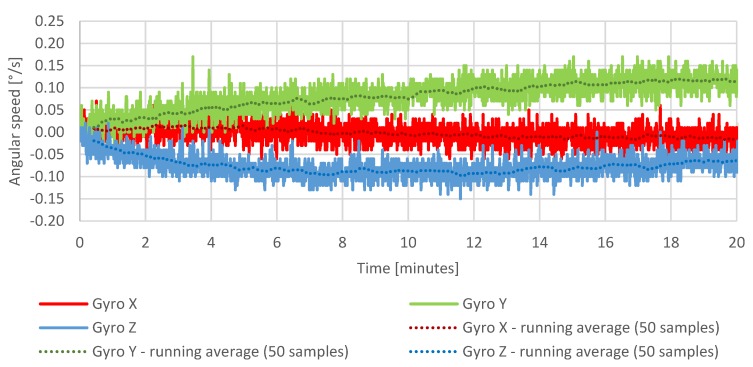
Stationary measurements of gyroscope drift.

**Figure 17:**
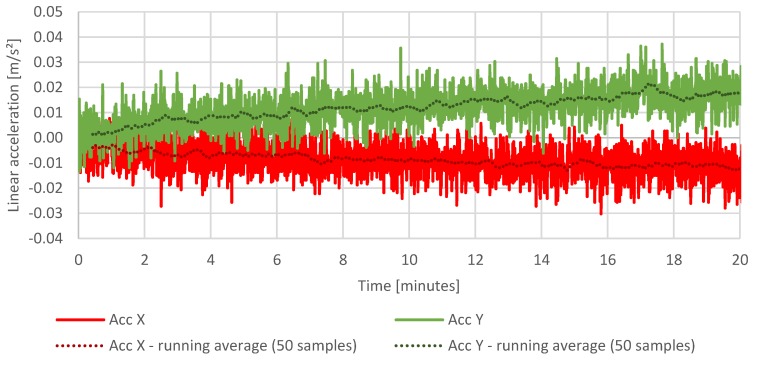
Stationary measurements of linear accelerations.

**Figure 18:**
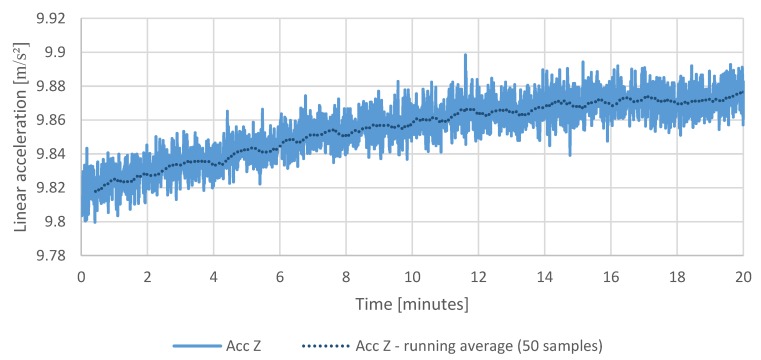
Stationary measurements of linear acceleration in the sensor vertical axis.

**Figure 19:**
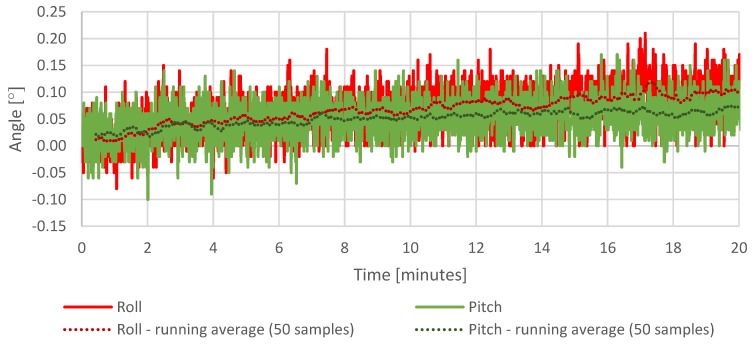
Stationary measurements of estimated roll and pitch angles.

**Figure 20:**
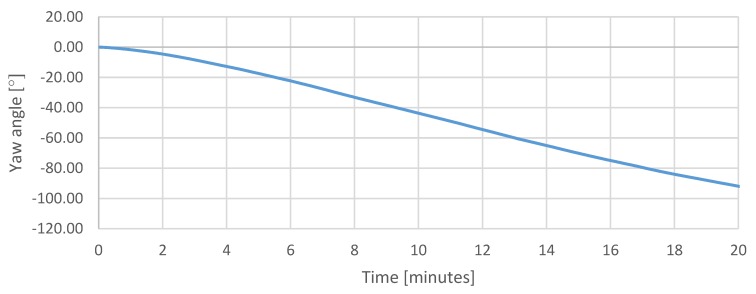
Stationary measurements of estimated yaw angle.

The first test is a static test showing the temperature of the measurement system, drift of the sensors, and the cumulating error of the attitude calculation in the same period of operation. The purpose of the test was to determine a relation between the temperature of the system and drift of the sensors and the attitude calculation error resulted from the drift in time. The IMU temperature throughout the test increased by approximately 10 °C (Figure 15), which had a visible impact on the sensor readings taken during that time (Figure 16, Figure 17 and Figure 18), which resulted in estimated roll and pitch angle values drifting from their initial values proportionally to the increasing sensor temperature (Figure 19 and Figure 20).

Figure 15 shows the increase in the temperature of the measurement system in 20 min test.

Figure 16 shows the increase of the gyroscopes drifts in 20 min test following the increase of the temperature of the measurement system.

Figure 17 and Figure 18 show the changes in reading of the accelerometers in axis x, y, z during 20 min test following the changes in temperature of the measurement system.

Figure 19 and Figure 20 show a cumulating error in calculated attitude. The pitch and roll calculated values increase in time, and the yaw calculated value decrease while the platform stays fixed.

The gathered readings show that the effectiveness of calculated offsets is correlated with the sensor temperature at the time of calibration. On the other hand, the estimated angle variations in roll and pitch are relatively small—up to +0.10 degrees deviation in roll and +0.05 in pitch, respectively (Figure 19).

The most affected sensor axis is the vertical axis responsible for tracking the yaw angle. Since the magnetometer was not yet implemented at the time of this test, whenever the sensor was operating horizontally to the ground, the yaw angle estimation relied solely on gyroscope measurements, which resulted in a high contribution of drift in the estimated rotation.

In practice, with aerial navigation not being one of the goals of the project, this issue could be neglected, especially in case of manual piloting, since it could be easily compensated using the trim function of the radio receiver. However, it does not diminish the need for proper temperature compensation in the future.

### 3.2. Controller Gains Examination

The second test was the controller step response test to the angular position step change with various values of the controller gain parameter. The purpose of this test was to determine the step response of the controller to the input angular movement exerted consecutively in each axis with various gain values.

The results of controller validation tests with selected gain settings are presented below. The results were used to evaluate both the validity of controller gains obtained using the Ziegler Nichols method and the response of implemented controllers to the introduced gain adjustments.

**Figure 21:**
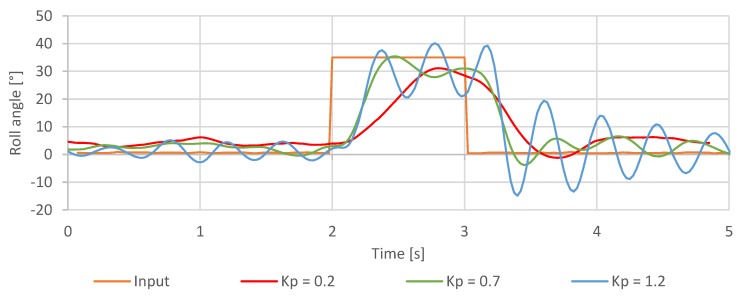
Step response of the roll axis controller for different Kp values.

The proportional gain was determined to be the most crucial for the operation of the inner loop controller. After decreasing the $K_{p\;rate\;roll}$ value, the quadcopter reactions became slower and less responsive (Figure 21). Since the output is directly proportional to the input error multiplied by the $K_{p\;rate\;roll}$ gain, the motors did not generate enough power required to position the quadcopter at the desired angle. On the other hand, after increasing the $K_{p\;rate\;roll}$ beyond the value of $K_{p\;rate\;roll}=0.9$, steady oscillations were observed with its amplitude increasing proportionally, which is a typical example of an overshoot. On that basis, it was determined that $K_{p\;rate\;roll}\approx 0.66$, calculated using the Z–N method, is close to optimal.

**Figure 22:**
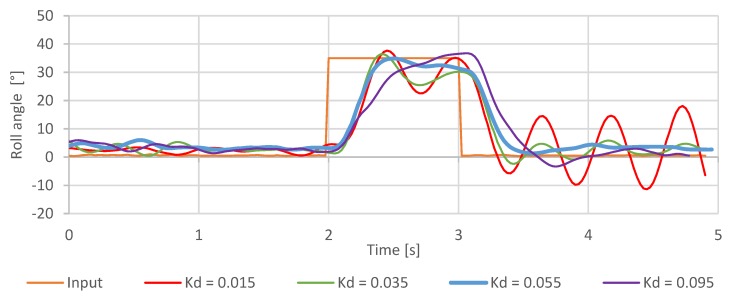
Step response of the roll axis controller for different Kd values.

The derivative gain evaluation was performed similarly. After evaluating the data (Figure 22), it was noted that derivative gain improves the overall system precision. It does that by reducing the overshoot and increasing the overall stability. This effect can be observed during flight—with higher $K_{d\;rate\;roll}$ values, the aircraft became “stiffer” and more resilient to air disturbances. During the stationary tests, $K_{d\;rate\;roll}=0.055$ turned out to be the optimal setting, which is a slight deviation from the results of the Z–N tuning.

Further experimentation showed that the range of useful derivative gain settings is much more restrictive when compared to proportional gain. Values reaching beyond $K_{d\;rate\;roll}=0.075$, caused derivate gain to display its dampening properties, by slowing down the quadcopter reaction to the pilot’s input. Increasing the $K_{d\;rate\;roll}$ even further—beyond the value of $K_{d\;rate\;roll}=0.110—$ introduced rapid oscillations in the rotation of the aircraft, with their frequency being much higher compared to the overshoot recorded during proportional gain tests. In this case, an over-amplification of the derivative gain causes the motor response with much more energy up to a point where the aircraft becomes uncontrollable.

**Figure 23:**
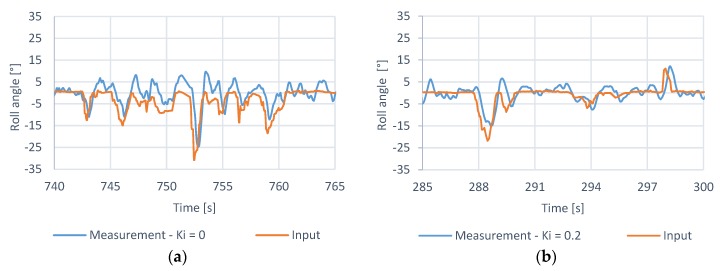
Hover stability with different Ki values, (**a**) measurement—Ki = 0, (**b**)—measurement—Ki = 0.2.

The value of the $K_{i\;}$ was tuned by testing a controller response to the random input roll angle changes demanded by the operator. The purpose of this test was to determine a response of the controller to the dynamically changing demanded angular position command with various values of the $K_{i}$ coefficient. An analysis of the data gathered during the hover stability test showed that $K_{i\;rate\;roll}$ has a noticeable impact on the quadcopter performance during hover. By disabling this gain, the quadcopter became more prone to drifting away from its takeoff location and required constant corrections from the pilot to stabilize its position. We expected this behavior as the purpose of the integral component is to nullify the steady-state error, which was mostly responsible for the drift. After increasing the integral gain value to $K_{i\;rate\;roll}=0.2$ the quadcopter stability improved as the desired angular position was maintained more effectively (Figure 23a,b). The integral gain also showed more resilience to overshoot as steady oscillations appeared beyond the value of $K_{i\;rate\;roll}=1.5$. This result, however, does not diminish the importance of anti-windup or other similar and potentially applicable safeguards as this test was conducted with the quadcopter being in-flight, during which the system was fully capable of maintaining its cumulated error within correctable boundaries.

As planned, the final adjustment to the PID gains was conducted between flights based on the observation of the quadcopter response to the pilot’s input and hover stability. The inner loop parameters were slightly re-adjusted to suit the pilot’s preferences better. The full set of final controller gains is presented in Table 3.

Following the tuning procedure, a series of tests were conducted to verify its efficacy. The trials were performed in flight, with a fully enabled controller set.

### 3.3. Control Loop Performance Test

The control loop performance test was a test of the stability of the drone. The attitude of the drone in relation to the demanded null value of angular angles pitch and roll was recorded. The purpose of this test was to determine the response of the controller to the natural disturbance during hover.

**Figure 24:**
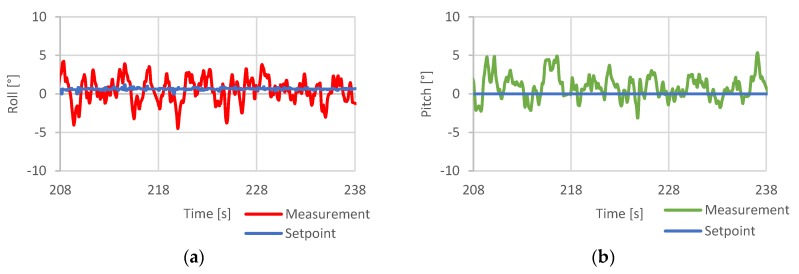
Hover stability test results, (**a**)—roll axis response, (**b**)—pitch axis response [5].

Figure 24 contains the hovering stability test results, during which the quadcopter was given null target roll and pitch angles to examine its ability to maintain horizontal orientation during hover flight.

The results proved that in its current state and applied settings, the developed system could achieve horizontal angular accuracy of ± 4° during hover flight. Due to the quadcopter high inertia, such oscillation had little effect on its position across the accounted timespan.

Next, the response time was verified in similar environmental conditions by applying a series of rapid maneuvers to register any significant delays between the pilot’s commands and their execution by the flight control system. The purpose of this test was to measure a delay of the drone position changing after the angular position change command receiving.

**Figure 25:**
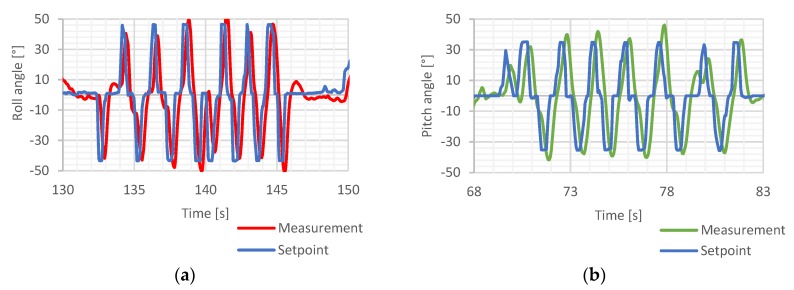
System response time test—$K_{u}=1.1$, (**a**)—roll axis, (**b**)—pitch axis [5].

The results of control loop response time evaluation test are presented in Figure 25.

The system response delay was measured to be around 200 ms. This result was considered acceptable since it can hardly be observed with the naked eye. The quadcopter behavior was appropriate, considering its takeoff mass and the use of large propellers.

### 3.4. Wi-Fi Telemetry Range Test

Wi-Fi telemetry range test was a test of successfully transferred data packets along with the increasing distance between the ground control station and the drone. The purpose of this test was to determine the maximum efficient range of the test platform telemetry.

**Figure 26:**
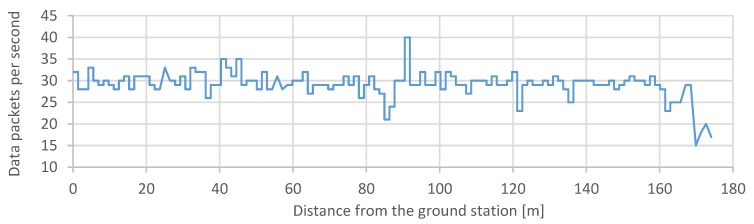
Wi-Fi telemetry system maximum operating range test [5].

Figure 26 shows the number of data packets from the quadcopter received by the ground station to the distance between network devices.

The maximum operation range of the ESP32 chip used in the flight controller construction was rated as approximately 150 m. This outcome is satisfactory, based on the fact that this functionality utilizes a miniature transmitter integrated within the chip structure. The device is well fit for this project use-case, being that of a close-range data transmission system.

## 4. Discussion

The custom quadcopter frame, with its foldable arms design, turned out to be a beneficial asset suitable for experimenting with custom avionics.

The proposed solution provides a tester with a mechanical part, which is a 3D printed frame with off-the-shelf motors and regulators. The frame is easily transportable and serves its purpose of protecting the electronic equipment mounted inside its central hub. In case of permanent damage, the parts can be quickly reprinted and replaced. This design could be modified in the future, preferably to reduce its size and mass while keeping the present functionality mostly intact.

The electrical part is ESP32 based flight controller with sensors and a software part with a code delivered for customization. The main chip of the flight controller, ESP32, turned out to be fast and efficient enough to complete the given tasks. The microcontroller, along with openly available libraries proved to be fully featured for flight controller applications. Its easily accessible wireless transmission capabilities make it a handy device for experimentation and data gathering, with some of its features remaining to be explored, the most prominent one being its multi-threading capabilities.

All the elements come together with a manual for the implementation of the users’ algorithms into the software code. In this way, the platform can be a reasonable solution for testing the control system algorithms at the initial stage of the UAV development, when the physical components are still not ready, and there is the need for the control and navigation algorithm testing against the requirements. Furthermore, the proposed platform can also find applications during the whole UAV lifecycle to face the need for the control algorithms upgrade. The idea of a customizable platform with the software open for implementing new algorithms allows testing various solutions in a short time. Moreover, the platform, thanks to its fast-folding frame, turns out to be highly portable, allowing the tests to be performed in a remote area.

The designed example control loop performed with satisfactory results considering its simplistic sensor input. The project suffered from initial drawbacks related to the underestimation of the influence of motor vibrations affecting the quadcopter’s estimated orientation, which resulted in the platform’s inability to maintain stable flight and occasional loss of control. Fortunately, the problem was reduced to tolerable levels with the help of an inertial sensor’s integrated low pass filter and through providing the sensor’s mounting point with additional isolation. The appropriate sensor configuration was a starting point for an example controller tuning process. The example algorithm of the cascade PID controller was developed and tested on the platform in consecutive steps described in the paper. Eventually, the process of tuning and testing resulted in a controller algorithm allowed for achieving the performance similar to that of off-the-shelf platforms of this type and size.

Since the software was designed with openness in mind, further hardware could be integrated without restrictions. The future addition of a magnetometer, barometer, and a GPS receiver could further increase the quadcopter capabilities with the potential introduction of essential flight autonomy.

**Figure 27:**
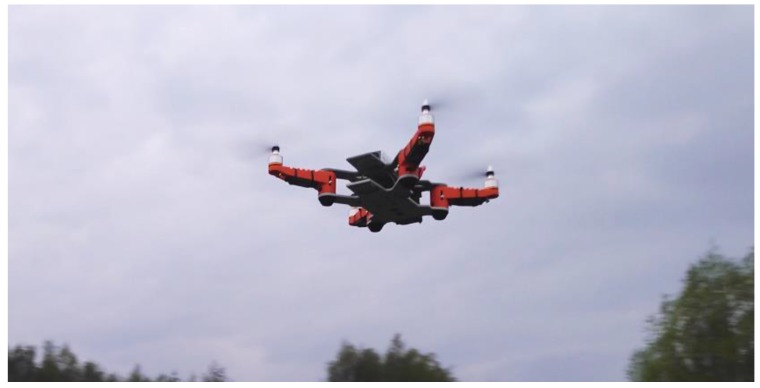
The quadcopter during flight.

The tuning method based on the Ziegler–Nichols approach proved to be sufficient in this example case, with minor corrections applied to the controller gain settings during flight tests to suit better the pilot’s flight style (Figure 27).

With its current sensor package, MPU6050, combined with the Madgwick’s AHRS filter, the quadcopter was fully capable of performing the flight using developed algorithms. The accuracy of this system, which directly relates to flight stability, could be increased with better vibration dampening and sensor temperature compensation. The latter should be feasibly done by mapping the changes in offset values together with recorded temperature readings and generating an approximated function through this series of data points. This function could then be integrated into the software to be used for calculating the current offset based on the recorded sensor temperature. By repeating this procedure for every sensor axis of both gyroscopes and accelerometers, it should be possible to mitigate the effects of temperature variations on the sensor readings.

The project has been successfully completed, and the initial goals have been achieved. A result is a functional medium-sized, customizable and easily accessible aerial platform, designed for experimental purposes. The task included the construction of the quadcopter itself, flight controller design and execution, control loop design, software development, and a practical implementation of the proposed example tuning procedure, used for the control loop setup.

The control loop tuning method, which was applied as an example, proved adequate to the point of providing controller gains, which enabled the quadcopter to take flight. The acquired settings were verified and tested during the series of trials, which confirmed their correctness. The platform with integrated diagnostic software turned out an appropriate tool for stabilization and control algorithm tuning.

The project also introduced a successful implementation of a short-range wireless telemetric data transmission module, integrated into the flight controller’s main processing chip, which further enhanced the platform’s capabilities and provided an additional source of research data during the project’s evaluation phase.

## Figures and Tables

**Table 1:** Full set of control loop adjustable parameters.

Axis	Inner Loop			Outer Loop		
roll	$P_{rate\;roll}$	$I_{rate\;roll}$	$D_{rate\;roll}$	$P_{angle\;roll}$	$I_{angle\;roll}$	$D_{angle\;roll}$
pitch	$P_{rate\;pitch}$	$I_{rate\;pitch}$	$D_{rate\;pitch}$	$P_{angle\;pitch}$	$I_{angle\;pitch}$	$D_{angle\;pitch}$
yaw	$P_{rate\;yaw}$	$I_{rate\;yaw}$	$D_{rate\;yaw}$	-	-	-

**Table 2:** Offset values of MPU6050 used in the flight controller.

	X-axis	Y-axis	Z-axis
**Gyroscope [°/s]**	34.58	33.88	37.26
**Accelerometer [m/s^2^]**	−0.37	10.61	−25.82

**Table 3:** Final control loop settings.

Axis	Inner Loop			Outer Loop		
	$K_{p\;rate}$	$K_{i\;rate}$	$K_{d\;rate}$	$K_{p\;angle}$	$K_{i\;angle}$	$K_{d\;angle}$
roll	*0.7*	*0.15*	*0.035*	*4.0*	*0.0*	*0.0*
pitch	*0.75*	*0.20*	*0.040*	*4.0*	*0.0*	*0.0*
yaw	*3.0*	*0.0*	*0.0*	*-*	*-*	*-*

## Appendix A

PID regulator implementation—the following method calculates and returns the regulator output value

float PID::Compute(float mSetpoint, float mInput) { float mError = mSetpoint - mInput; // input error value unsigned long tn = millis(); // current time in [ms] float dt = (float)(tn - tp); // step interval float P = (float)(kp * mError); // P gain float I = (float)(Ip + ki * mError * dt / 1000.0); // I gain float D = (float)(-1 * (kd * (mInput - pInput) * 1000.0 / dt)); // D gain (measurement derivative) if (anti_windup) // anti-windup (clearing the integral value) { I = 0; } float U = P + I + D; // sum of all components pInput = mInput; // history pError = mError; Ip = I; tp = tn; return U; // output value }
